# Age- and Severity-Associated Humoral Immunity Response in COVID-19 Patients: A Cohort Study from Wuhan, China

**DOI:** 10.3390/jcm11195974

**Published:** 2022-10-10

**Authors:** An Zhu, Min Liu, Yang Li, Qing Lei, Qiaoyi Wu, Mingxi Lin, Danyun Lai, Linfang Lu, Siqi Yu, Shujuan Guo, Hewei Jiang, Hongyan Hou, Yunxiao Zheng, Xuening Wang, Mingliang Ma, Bo Zhang, Hong Chen, Junbiao Xue, Hainan Zhang, Huan Qi, Ziyong Sun, Feng Wang, Xionglin Fan, Shengce Tao, Zhaowei Xu

**Affiliations:** 1 Key Laboratory of Gastrointestinal Cancer (Fujian Medical University), Ministry of Education, Fuzhou 350122, China; 2 Laboratory of Scientific Research, School of Basic Medical Sciences, Fujian Medical University, Fuzhou 350122, China; 3 Fujian Key Laboratory of Tumor Microbiology, Department of Medical Microbiology, Fujian Medical University, Fuzhou 350122, China; 4 Shanghai Center for Systems Biomedicine, Key Laboratory of Systems Biomedicine (Ministry of Education), Shanghai Jiao Tong University, Shanghai 200240, China; 5 Department of Pathogen Biology, School of Basic Medicine, Tongji Medical College, Huazhong University of Science and Technology, Wuhan 430074, China; 6 The Trauma Center and Emergency Surgery, Department of the First Affiliated Hospital of Fujian Medical University, Fuzhou 350004, China; 7 Department of Clinical Laboratory, Tongji Hospital, Tongji Medical College, Huazhong University of Science and Technology, Wuhan 430074, China

**Keywords:** SARS-CoV-2, protein microarray, age, humoral immunity

## Abstract

Age has been found to be the single most significant factor in COVID-19 severity and outcome. However, the age-related severity factors of COVID-19 have not been definitively established. In this study, we detected SARS-CoV-2-specific antibody responses and infectious disease-related blood indicators in 2360 sera from 783 COVID-19 patients, with an age range of 1–92 years. In addition, we recorded the individual information and clinical symptoms of the patients. We found that the IgG responses for S1, N, and ORF3a and the IgM for NSP7 were associated with severe COVID-19 at different ages. The IgM responses for the S-protein peptides S1-113 (aa 673–684) and S2-97 (aa 1262–1273) were associated with severe COVID-19 in patients aged <60. Furthermore, we found that the IgM for S1-113 and NSP7 may play a protective role in patients aged <60 and >80, respectively. Regarding clinical parameters, we analyzed the diagnostic ability of five clinical parameters for severe COVID-19 in six age groups and identified three-target panel, glucose, IL-6, myoglobin, IL-6, and NT proBNP as the appropriate diagnostic markers for severe COVID-19 in patients aged <41, 41–50, 51–60, 61–70, 71–80, and >80, respectively. The age-associated severity factors revealed here will facilitate our understanding of COVID-19 immunity and diagnosis, and eventually provide meaningful information for combating the pandemic.

## 1. Introduction

COVID-19, caused by SARS-CoV-2, has become a worldwide pandemic and poses a great threat to public health. Globally, by 25 February 2022, there had been 431,415,000 confirmed cases of COVID-19, resulting in 5,928,000 deaths. Mild symptoms of COVID-19, such as fever, cough, and fatigue, appear after infection [1]. However, severe COVID-19 can cause serious complications, such as organ injury and an increased risk of death [2]. Hence, identifying severity-associated factors is conducive to the treatment of COVID-19 patients and to fighting the pandemic [3].

Age is the single greatest factor in COVID-19 severity and outcome [4,5]. The mortality is <1% in patients aged <50 years, but it increases exponentially with age. The highest mortality was observed in patients ≥80 years of age [6,7]. The risk of severe COVID-19 differs considerably according to age, perhaps due to complex factors [8,9,10]. Therefore, the systematic exploration of severity-related factors in different age groups will be of great significance for the precise diagnosis and treatment of COVID-19.

Humoral immune responses, especially SARS-CoV-2-specific antibody responses, play critical roles in the disease’s progression, severity, and final outcome [11,12]. S-protein-specific antibody responses are correlated with neutralization activity against the SARS-CoV-2 virus [13]. IgG responses against non-structural/accessory proteins, i.e., NSP1, NSP7, and ORF9b, are associated with the severity of COVID-19 [14]. In addition, the levels of IgG antibodies against NSP4 and ORF3b have a predictive power for patient mortality [15]. However, the correlation analysis between COVID-19-specific antibody responses and severity did not consider age as a variable.

Clinical serum indicators have been used in the prediction and diagnosis of severe COVID-19, and they have been widely verified in clinical settings [16,17]. IL-6 is a biomarker for the development of severe COVID-19 [18], and it has been exploited as a potential cytokine target for therapy [19]. In addition, IL-2R and TNF-α were found to predict COVID-19 severity and survival [17]. However, it is unclear whether the potential diagnostic and therapeutic effects are consistent at different ages.

In this study, we performed a comprehensive analysis of the SARS-CoV-2-specific immune responses in 783 COVID-19 patients. We identified the severity-associated factors, such as SARS-CoV-2-specific antibody responses and clinical parameters for COVID-19 patients of different ages.

## 2. Materials and Methods

### 2.1. Hospitalized COVID-19 Patients

All 783 patients which entered the cohort from hospitalization in Tongji Hospital in Wuhan, China, included 387 males and 396 females, and the mean age was 63.4 years, with an age ranging from 1 to 92 years. Ethical approval was provided by the Ethical Committee of Tongji Hospital, Huazhong University of Science and Technology, China (REC reference: ITJ-C20200128). COVID-19 patients who meet any of the following conditions were diagnosed as severe: (1) respiratory distress (≥30 breaths/min), (2) oxygen saturation ≤ 93% at rest, (3) arterial partial pressure of oxygen (PaO_2_)/fraction of inspired oxygen (FiO_2_) ≤ 300 mmHg, and (4) chest imaging that showed obvious lesion progression (>50%) within 24–48 h. Serum samples were collected from hospitalized COVID-19 patients and stored at −80 °C.

### 2.2. SARS-CoV-2 Proteome Microarray Construction

The SARS-CoV-2 proteome microarray contained the 21 SARS-CoV-2 proteins [20] and the 197 S-protein peptides [21], and was generated in the 14-subarray format on PATH slides (Grace Bio-Labs, Bend, OR, USA) using a Super Marathon printer (Arrayjet, Roslin, UK). ACE2 protein, human IgG, human IgM, and GST protein were added to the microarray as controls.

### 2.3. SARS-CoV-2-Specific Antibody Response Analysis

The SARS-CoV-2 proteome microarray was used for serum profiling to obtain SARS-CoV-2-specific antibody responses. The profiling methods were described previously [22]. Briefly, the arrays were warmed to room temperature and incubated in blocking buffer (3% BSA in 1× PBST, 0.1% Tween 20 was added unless otherwise noted) for 3 h. A 14-cavity rubber gasket was mounted on each slide, creating individual cavities for 14 identical subarrays. Each subarray was cocultured with 200 μL of diluted serum for 2 h. Arrays were washed with 1× PBST 6 times, and the bound antibody was monitored by incubating the arrays with Cy3-conjugated anti-human IgG and Alexa Fluor 647-conjugated anti-human IgM (Jackson ImmunoResearch, West Grove, PA, USA) for 1 h at room temperature. The microarrays were washed with 1× PBST 6 times and centrifuged at room temperature to dry them. The IgM and IgG intensities were determined using a LuxScan Scanner (CapitalBio, Beijing, China). The fluorescence intensities were extracted using the GenePix 6.0 software (Molecular Devices, San Jose, CA, USA).

### 2.4. SARS-CoV-2 Proteome Microarray Data Analysis

The raw GPR microarray data were read by limma in R. The IgG (532 fluorescent channel) and IgM (635 fluorescent channel) intensities were defined as the median foreground (F) minus the median background (B) for each spot, and the protein signal intensities of three replicate spots were averaged. Block #14 of each slide was incubated with SARS-CoV-2 immunopositive serum as a positive control. For microarray data normalization, the normalization factor was calculated for each slide by linear regression based on the positive control and data were normalized between slides using a linear approach to positive controls. To reduce errors between microarrays, the signals for all proteins on each slide were segmented by their normalization factors.

### 2.5. The Collection of Clinical Parameters

The clinical parameters associated with infectious diseases were collected from Tongji Hospital, Wuhan, China.

### 2.6. Quantification and Statistical Analysis

The data were analyzed by using SPSS 24.0 software (IBM, New York, NY, USA) and expressed as the mean ± standard deviation (SD) for continuous variables and as the frequency (%) of categorical variables. Comparisons between groups were performed using the chi-squared test for the categorical variables and the two-sided *t*-test for the continuous variables. The logistic regression analysis was performed to assess the odds ratios (ORs) and 95% confidence intervals (CIs) for the associations between outcomes (severity and overcome) and different influencing factors (ages). Values of two-sided * *p* < 0.05, ** *p* < 0.01, or *** *p* < 0.01 were considered statistically significant.

## 3. Results

### 3.1. Characteristics of the COVID-19 Patients in the Cohort

To obtain a more comprehensive understanding of the factors affecting the severity of and death from COVID-19, a total of 2360 sera from 783 patients diagnosed with COVID-19 from Tongji Hospital, Wuhan, China, were collected (Table 1). All the COVID-19 patients in this study were not vaccinated. The median age of the 783 patients was 61.4 years, and 48% of them were female. Among the 783 patients, there were 369 (47%) non-severe cases and 414 (53%) severe cases, of which 723 survived and 60 did not survive. Previous studies have shown that the severity will increase significantly if the patient is older than 40 years of age. To study the correlation between age and severity, we divided these patients into six groups by age, i.e., <41, 41–50, 51–60, 61–70, 71–80, and >80 (Table 2).

To understand the risks of severe disease and death in COVID-19 patients of different ages, we performed a statistical comparison among the groups. Previous studies have shown that gender has a considerable effect on the severity and outcome of COVID-19 [23,24]. In this study, we found that males had a higher risk of severe COVID-19 than females among patients aged 51–80 (Table S1). Hence, we analyzed the logistic regression parameter of severity in association with age and adjusted it for gender. The results show that the Wald χ^2^ of the logistic regression parameter for severity decreased from 36.736 to 0.302 as age increased and the OR of severe risk decreased from 1 to 0.123 as age decreased. The survival-related Wald χ^2^ decreased from 12.264 to 3.953 as age increased, and the OR for death decreased from 1 to 0.066 as age decreased (Table 3). The results show, in this cohort, that age is a risk factor for severe disease and death in COVID-19 patients, which is consistent with previous reports [4,25].

To understand the role of medical history in COVID-19, we performed the binary logistic regression parameter of severity and outcome in association with the medical history among COVID-19 patients (Table S2). The results show that hypertension, diabetes mellitus, cardiovascular disease, and cancer are associated with the severity of COVID-19, and cardiovascular disease and cancer are associated with the outcome of COVID-19. Hypertension, diabetes mellitus, cardiovascular disease, and cancer are closely related to age. It is said that medical diseases are age-related severe COVID-19 factors.

The landscape of severity-associated SARS-CoV2-specific antibody responses and clinical parameters in different age groups is shown in Table 4.

To obtain SARS-CoV-2-specific antibody responses, we screened 2360 serum samples using a SARS-CoV-2 proteome microarray [26], which contained 21 SARS-CoV-2 proteins and 197 S-protein peptides with full coverage of the SARS-CoV-2 spike protein. Furthermore, 96 blood indicators associated with infectious diseases were also measured, e.g., IL-6, IL-2R, and d-dimer.

The serum samples were collected at ~30 days after the onset of symptoms, at which time the IgM and IgG responses were relatively stable. We indicated the time of onset to sampling and the data showed that there was no significant difference among patients of different ages (Figure S1A). To compare the antibody response in patients of different ages, we performed UMAP analysis of IgG and IgM responses, and the results show that the six groups of patients were dispersedly distributed on the coordinate axis and had no significant clustering (Figure S1B). In addition, to more intuitively compare the IgG/IgM levels of patients of different ages, we constructed response landscape maps of IgG and IgM for 21 SARS-CoV-2 proteins (Figure S1C). The results show that the IgG response of SARS-CoV-2 proteins are positively correlated with age.

To explore the severity-associated SARS-CoV-2-specific antibody responses and clinical features, we compared the mild and severe cases of COVID-19 in the six groups at three levels: protein responses, S-protein peptide responses, and clinical parameters. First, the parameters, e.g., antibody responses and clinical parameters, were divided into six groups by age. Second, in each age group, we calculated whether the parameters of mild and severe patients were significantly different. A two-sided *t*-test was used for the significance calculation. The results show that the responses to four proteins (N-protein IgG, S1 IgG, ORF-3a IgG, and NSP7 IgM), 10 peptides, and 28 clinical parameters were associated with disease severity at the respective ages (Table 4). The severity-associated SARS-CoV-2-specific antibody responses and clinical features significantly varied among the age groups.

### 3.2. Severity-Associated SARS-CoV-2-Specific Antibody Responses in Different Age Groups

**Figure 1:**
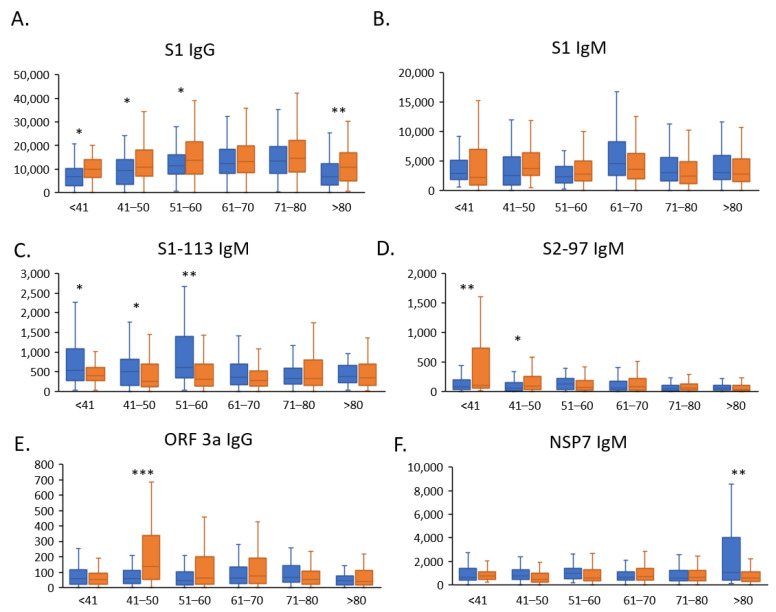
The severity-associated SARS-CoV-2-specific antibody responses in different age groups. The histogram shows the antibody responses for the spike protein (**A**) and N protein (**B**), spike protein peptides (**C**,**D**), and nonstructural proteins ORF 3a (**E**) and NSP7 (**F**) in COVID-19 patients with mild to severe disease in different age groups. Blue histograms represent mild patients and orange histograms represent severe patients. Blue = mild patients; Yellow = severe patients. * *p* < 0.05, ** *p* < 0.01, or *** *p* < 0.01 were considered statistically significant.

We then visualized the antibody responses using a histogram and found that the S1 IgG responses were significantly increased in severe patients aged <60 years (*p* < 0.05) and more significantly increased in patients aged >80 years (*p* < 0.01), while no significant differences were observed in patients aged 60–80 years (Figure 1A). The S1 IgM responses were not significantly different in severe COVID-19 patients (Figure 1B). To study the antibody response for each epitope on the S-protein, we compared the mild and severe cases of COVID-19 for the S-protein peptide responses. The IgM responses for S2-97, a highly immunogenic linear epitope, were significantly increased in severe patients <50 years of age and had the same trend as the S-protein responses (Figure 1C). In addition, the S-protein peptide S1-113 (673–684 aa) is on the outer surface of the S1/S2 cleavage site and was identified as a highly immunogenic epitope [27]. We found that the IgM responses of S1-113 were significantly decreased in severe patients <60 years of age (Figure 1D).

Nonstructural proteins of SARS-CoV-2 play key roles in virus replication and immune escape, i.e., ORF 3a is involved in NLRP3 inflammation activation [28] and NSP7 forms a complex with NSP8 to enhance RdRp activity [29]. We found that the ORF-3a IgG responses were significantly increased in severe patients aged 40–50 years (Figure 1E). Conversely, the NSP7 IgM responses were significantly decreased in severe COVID-19 patients aged >80 years (Figure 1F).

### 3.3. Antibody Responses for S1-113 IgM and NSP7 IgM Are Protective Factors for Severe COVID-19 Patients

To explore the function for severity-associated antibody responses, we selected the severe COVID-19 patients with high antibody responses of S1 IgG, S1-113 IgM, ORF-3a IgG, NSP7 IgG, and S2-97 IgM, and then analyzed the correlation between antibody responses and severity (non-critical/critical), outcomes (survivor/non-survivor). The results show that, in severe COVID-19 patents, higher S1 IgG, S2-97, and ORF 3a IgG responses are not correlated with severity or outcome. However, the severe patients with higher S1-113 IgM or NSP7 IgM responses had milder symptoms and a lower mortality rate (Table 5). In addition, we found that the responses of S1-113 IgM were higher in mild COVID-19 patients than in severe patients aged <60 years (Figure 1C), and the responses of NSP7 IgM were higher in mild COVID-19 patients than in severe patients aged >80 years (Figure 1F). Hence, S1-113 IgM and NSP7 IgM may play a protective role in patients aged <60 and >80 years, respectively.

### 3.4. The Severity-Associated Clinical Parameters in Different Age Groups

**Figure 2:**
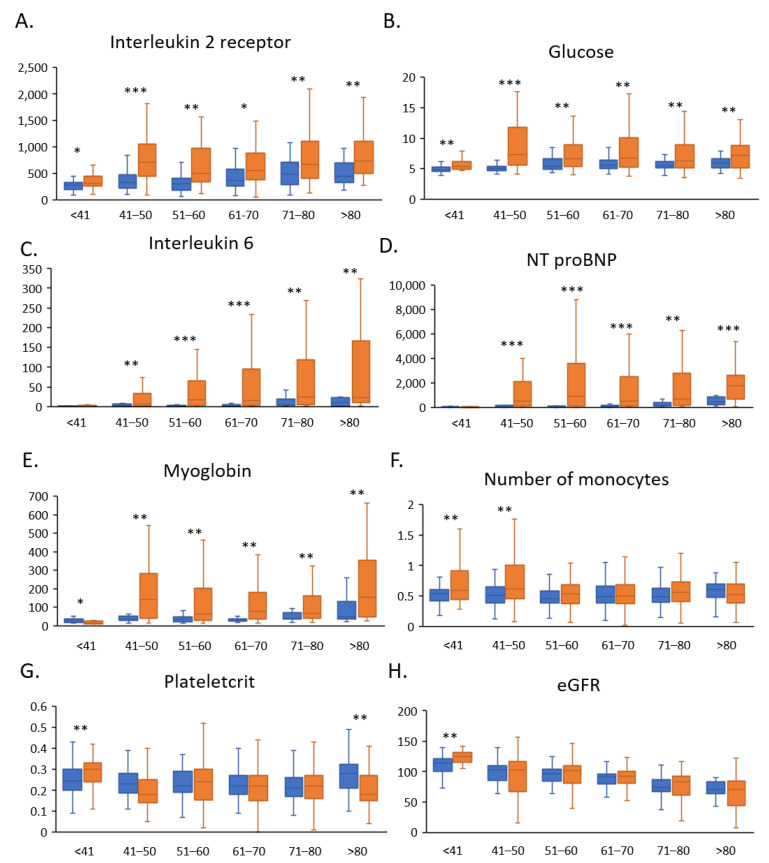
COVID-19 patient clinical parameters in different age groups. The histogram shows clinical parameters in COVID-19 patients with mild to severe disease in different age groups, i.e., (**A**) Interleukin 2 receptor, (**B**) Glucose, (**C**) Interleukin 6, (**D**) NT proBNP (N-terminal pro brain natriuretic peptide), (**E**) Myoglobin, (**F**) Number of monocytes, (**G**) Plateletcrit, (**H**) eGFR. Blue histograms represent mild patients and orange histograms represent severe patients. Blue = mild patients; Yellow = severe patients. * *p* < 0.05, ** *p* < 0.01, or *** *p* < 0.01 were considered statistically significant.

To better understand the different pathogeneses and complications in COVID-19 patients of different ages, we compared the clinical parameters in different age groups between mild and severe patients. We observed a significant increase in interleukin 2 receptor (IL-2R) and glucose levels in the blood of severe COVID-19 patients in all age groups (Figure 2A,B). This result is consistent with the findings of Hou et al. [30]. In addition, high levels of interleukin 6 (IL-6), NT-proBNP, and myoglobin were associated with the development of more severe disease in previous studies [31,32,33]. Our results show that IL-6, NT-proBNP levels significantly increased in severe patients >41 years of age, while Interleukin 6, NT-proBNP levels were not significantly different in severe patients <40 years of age (Figure 2C–E).

Monocytes are a part of the innate immune system and play an important role in antiviral immunity. We found that the monocyte counts in the blood significantly increased in severe COVID-19 patients <50 years of age (Figure 2F). Plateletcrit (PCT) shows a negative correlation with the degree of inflammation in hepatitis infection [34]. We found that PCT significantly increased in severe COVID-19 patients aged <40 years and decreased in severe patients aged >80 years (Figure 2G). In addition, patients with COVID-19-associated acute kidney injuries exhibited a greater decrease in eGFR [35]. We found that the eGFR levels were significantly increased in severe patients <40 years of age (Figure 2H). These results imply that extremely young COVID-19 patients may show an activation of stronger antiviral immunity and have a higher risk of acute kidney injury.

Pathogen co-infection is one of the important factors for complications of COVID-19. Pathogen co-infection was detected in 217 (27.7%) patients: 121 patients had influenza viruses, 9 patients had mycoplasma pneumoniae, and 1 patient had Legionella pneumophila. We analyzed the co-infection of influenza viruses in different age groups. The results show that the co-infection of influenza viruses was enriched in mild COVID-19 patients aged <41 years (Table 6). However, this result needs to be verified by a larger sample.

### 3.5. The Clinical Parameters Have Different Diagnostic Abilities in Different Age Groups

**Figure 3:**
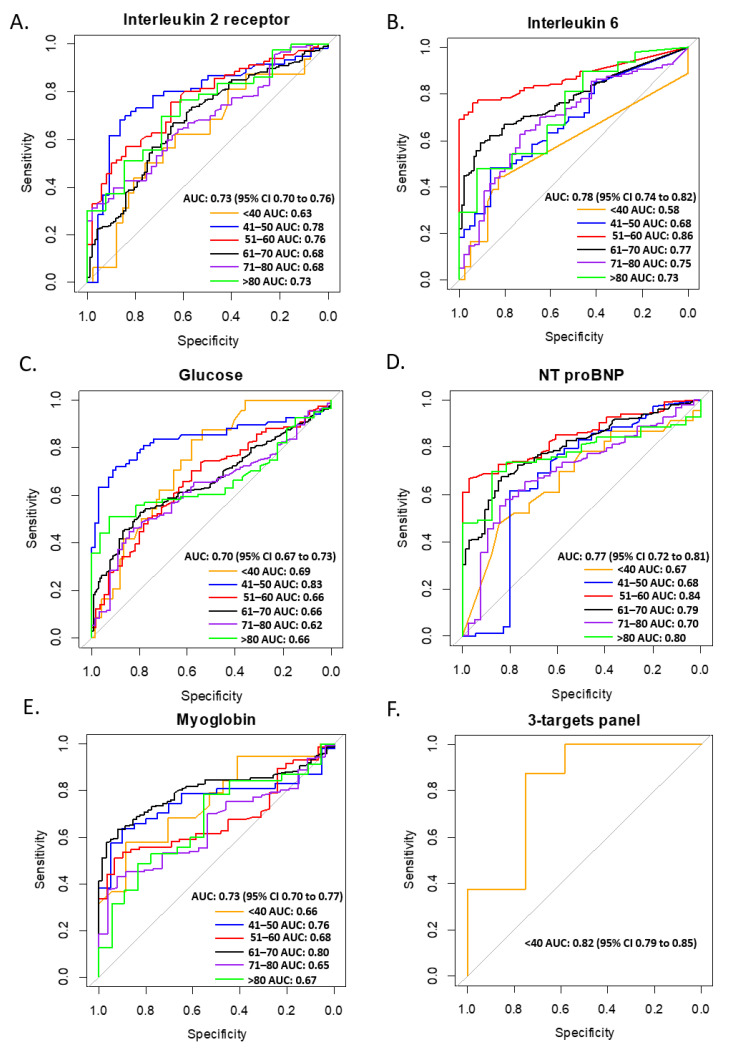
Receiver Operating Characteristic (ROC) curves for Interleukin 2 receptor (**A**), Interleukin 6 (**B**), glucose (**C**), NT proBNP (**D**), myoglobin (**E**), and three-target panel (**F**) for identifying individuals with severe COVID-19. AUC, area under the curve; CI, confidence interval; NT proBNP, N-terminal pro brain natriuretic peptide.

Several clinical laboratory parameters have been used for the prediction or diagnosis of severe COVID-19 [36,37]. However, the diagnostic ability of clinical parameters in different age groups has not been definitively established. Here, we analyzed the ROC of Interleukin 2 receptor, Interleukin 6, glucose, NT proBNP, and myoglobin for the diagnosis of severe COVID-19 in six age groups. The results show that the AUCs of Interleukin 2 receptor, Interleukin 6, glucose, NT proBNP, and myoglobin were 0.73 (95% CI: 0.70 to 0.76), 0.78 (95% CI: 0.74 to 0.82), 0.70 (95% CI: 0.67 to 0.73), 0.77 (95% CI: 0.72 to 0.81), and 0.73 (95% CI: 0.70 to 0.77), respectively (Figure 3). In different age groups, glucose had higher AUC in patients aged <41 years (0.69, 95% CI: 0.65 to 0.73) and aged 41–50 years (0.83, 95% CI: 0.79 to 0.86) (Figure 3C), Interleukin 6 had a higher AUC in patients aged 51–60 years (0.86, 95% CI: 0.83 to 0.90) (Figure 3B), myoglobin had a higher AUC in patients aged 61–70 years (0.80, 95% CI: 0.78 to 0.83) (Figure 3E), Interleukin 6 had a higher AUC in patients aged 71–80 years (0.75, 95% CI: 0.72 to 0.79) (Figure 3B), and NT proBNP had a higher AUC in patients aged >80 years (0.80, 95% CI: 0.78 to 0.82) (Figure 3D). To obtain a higher diagnostic ability in the aged <41 years group, we combined three parameters, i.e., Interleukin 2 receptor, glucose and myoglobin as the tree-target panel. The AUC of the tree-target panel was 0.82 (95% CI: 0.79 to 0.85) for the diagnosis of severe COVID-19 in patients aged <41 years (Figure 3F). Hence, the clinical parameters are age-specific for the diagnosis of severe COVID-19.

## 4. Discussions

In this study, we analyzed the SARS-CoV-2-specific antibody responses and the blood indicators in 2360 sera from 783 patients, and identified severity-associated factors among six age groups. Among the SARS-CoV-2-specific antibody responses, we found that those for S1-113 IgM and NSP7 IgM may play protective roles in patients aged <60 and >80 years, respectively. For the clinical parameters, we identified the appropriate diagnostic markers for each age group. Hence, we provided a systematic analysis of age-related factors for COVID-19 severity.

Spike protein antibody responses correlate with disease severity [38,39]. Our results show that S1 IgG responses were increased in severe patients <60 years of age and >80 years of age but not in severe patients <60–80 years of age. To explore this inconsistency, we divided the patients into three groups with low, medium, and high signals (25%, 50%, and 25%, respectively), and calculated the enrichment statistical analysis for the patients with high S1 IgG responses. The results show that high S1 IgG responses were enriched in the 60- to 80- year-old group (Table S3). Therefore, we propose that COVID-19 patients aged 60–80 years tend to have a higher S1 antibody response, and there is no difference between mild and severe patients. However, the potential immune mechanism remains to be explored.

In addition, we found that strong responses of S1-113 IgM and NSP7 IgM were correlated with severity and mortality in patients aged <60 and >80 years, respectively. In addition, the responses of S1-113 IgM and NSP7 IgM are higher in mild COVID-19 patients. Therefore, the results imply that S1-113 IgM and NSP7 IgM play protective roles in COVID-19 patients and may also be used for evaluating the effectiveness of vaccines.

Although advancing age is associated with a greater risk of death in both genders, the male bias remains evident. Previous studies have shown that gender has a considerable effect on the severity and outcome of COVID-19. In this study, we found that males had a higher risk of severe COVID-19 than females among patients aged 51–80 years. Multiple factors affect the risk of severe COVID-19 for different genders. First, Iwasaki et al. found key differences in the baseline immune capabilities, such as innate immune chemokines, cytokines, and T-cell responses in men and women during the early phase of a SARS-CoV-2 infection, which suggests distinct immune mechanisms of disease progression between the sexes [40]. Second, differences in pharmacology may be an important factor, but the information on sex-targeted treatment strategies is currently limited [41]. In our previous study, we found that ORF9b antibody responses and creatinine levels in the serum were associated with severity in male COVID-19 patients, which suggests differences in pathogeneses and complications between male and female COVID-19 patients [42].

IL-6 is an indicator of the severity of COVID-19, referencing the Diagnosis and Treatment Protocol for Novel Coronavirus Pneumonia (Trial Version 7). Our data show that the IL-6 level is increased in severe patients aged <40 years but is not significantly different in severe patients aged <40 years. In addition, Plateletcrit and eGFR were increased in severe patients aged <40 years. It is stated that the features of the clinical parameters are age-specific. Indeed, the data show that the AUCs of clinical parameters were greatly different in each age group. Therefore, defining the diagnostic ability according to age will improve diagnostic accuracy.

In the present study, age, gender, and severity were considered as important factors for the humoral immunity response of COVID-19 patients; therefore, age was stratified and gender was adjusted in the logistic regression model. According to the existing studies, obesity is also a vital factor involved in the outcome of COVID-19, usually measured by the body mass index which is calculated as weight in kilograms divided by height in meters squared [43,44,45]. A meta-analysis revealed that people with high obesity had higher risks of morbidity and mortality due to COVID-19 [46,47,48]. However, the height and weight data were not collected during the admission of the patients; thus, the role of BMI in COVID-19 could not be analyzed and became a defect of this study.

In conclusion, we revealed severity-associated SARS-CoV-2-specific antibody responses and clinical features in COVID-19 patients of different ages. The results show that four protein responses, i.e., N-protein IgG; S1 IgG; ORF-3a IgG; and NSP7 IgM, 10 S-protein peptide responses, and 28 clinical parameters were associated with disease severity at the respective ages. The comprehensive analysis of mild/severe patients of different ages may facilitate a deeper understanding of the pathogenesis and complications of SARS-CoV-2.

## Figures and Tables

**Table 1:** The clinical characteristics of the diagnosed COVID-19 patients.

Group		COVID-19 Case (n)
Patients (n)		783
Serum samples (n)		2360
Age (year)		61.4 ± 14.5
Gender	Male	377
	Female	379
Severity/outcome	Non-severe	369
	Severe	414
	Survivor	723
	Non-survivor	60
Source		Tongji Hospital, Wuhan

**Table 2:** The clinical characteristics of the diagnosed COVID-19 patients in different age groups.

	Symptom		Outcome		Patients (n)	Serum Samples (n)	Onset Time (d)	Gender	
	Mild	Severe	Cured	Death				Female	Male
<41	68	18	86	0	86	163	41 ± 17	37	49
41–50	56	34	88	2	90	249	52 ± 21	43	47
51–60	71	83	142	12	154	444	51 ± 18	79	75
61–70	106	133	223	16	239	713	52 ± 19	124	115
71–80	50	108	140	18	158	571	51 ± 18	83	75
>80	18	38	44	12	56	219	47 ± 19	30	26

**Table 3:** The logistic regression parameter of severity and clinical outcome in association with the age among diagnosed COVID-19 patients.

	Severity											Clinical Outcome								
	Crude Model					Gender-Adjusted Model						Crude Model				Gender-Adjusted Model				
Age	*β*	S.E.	Wald c2	OR (95% CI)	*p*	*β*	S.E.	Wald c2	OR (95% CI)	*p*	*β*	S.E.	Wald c2	OR (95% CI)	*p*	*β*	S.E.	Wald c2	OR (95% CI)	*p*
≤40	−2.01	0.34	34.84	0.13 (0.07, 0.26)	0.00	−2.10	0.35	36.76	0.12 (0.06, 0.24)	0.00	−2.68	0.77	12.04	0.07 (0.02, 0.31)	0.00	−2.71	0.77	12.26	0.07 (0.02, 0.30)	0.00
41–50	−1.24	0.32	15.33	0.29 (0.16, 0.54)	0.00	−1.31	0.32	16.65	0.27 (0.14, 0.51)	0.00	−2.36	0.66	12.94	0.09 (0.03, 0.34)	0.00	−2.39	0.66	13.24	0.09 (0.03, 0.33)	0.00
51–60	−0.86	0.29	8.69	0.42 (0.24, 0.75)	0.00	−0.86	0.3	8.44	0.42 (0.24, 0.76)	0.00	−1.17	0.4	8.75	0.31 (0.14, 0.67)	0.00	−1.16	0.4	8.56	0.31 (0.14, 0.68)	0.00
61–70	−0.53	0.28	3.58	0.59 (0.34, 1.02)	0.06	−0.53	0.29	3.45	0.59 (0.34, 1.03)	0.06	−1.19	0.37	10.38	0.30 (0.15, 0.63)	0.00	−1.19	0.37	10.23	0.31 (0.15, 0.63)	0.00
71–80	−0.11	0.3	0.14	0.90 (0.50, 1.61)	0.71	−0.09	0.3	0.09	0.91 (0.51, 1.65)	0.76	−0.76	0.38	4.08	0.47 (0.22, 0.98)	0.04	−0.75	0.38	3.95	0.47 (0.22, 0.99)	0.05
>80	1.00					1.00					1.00					1.00				

S.E., standard error; OR, odds ratio.

**Table 4:** The landscape of severity-associated SARS-CoV2-specific antibody responses and clinical parameters in different age groups.

Theme	Parameter	<41	41–50	51–60	61–70	71–80	>80
Protein responses	N-protein IgG						↑
	S1 IgG	↑	↑	↑			↑
	ORF 3a IgG		↑				
	NSP7 IgM						↓
S-protein peptide responses	S1 113 IgM	↓	↓	↓			
	S2 18 IgG			↑	↓	↑	
	S2 97 IgM	↑	↑				
	S2 96 IgM	↑				↑	
	S1 90 IgG				↓		↑
	S2 11 IgM				↑	↑	
	S2 15 IgM				↑	↑	
	S2 79 IgM					↓	↑
	S2 58 IgM	↓					
	S2 27 IgM	↓		↓			
Clinical parameters	Interleukin 2 receptor	↑	↑	↑	↑	↑	↑
	Platelet hematocrit	↑					↓
	Procalcitonin	↑	↑	↑	↑	↑	↑
	NT-proBNP		↑	↑	↑	↑	↑
	RBC distribution width SD	↑	↑	↑		↑	
	Albumin	↓	↓	↓		↓	
	Creatinine	↓		↓			↑
	Ferritin		↑	↑	↑		
	Glucose	↑	↑	↑	↑	↑	↑
	Phosphorus		↓	↓	↓		
	Platelet count		↓	↑	↓		↓
	TBIL 0.8		↑	↑			↑
	TNF			↑			
	Total Bilirubin		↑	↑			↑
	Glutamyl transpeptidase	↑	↑				
	Number of monocytes	↑	↑				
	Chlorine	↓	↓				
	High density lipoprotein				↓	↓	
	Lymphocyte					↓	↓
	PLT distribution width					↑	↑
	White ball ratio				↓	↓	
	eGFR	↑					
	Interleukin 6		↑	↑	↑	↑	↑
	Neutrophil count	↑					
	Myoglobin	↓	↑	↑	↑	↑	↑
	CRP						↑
	D-dimer						↑

ORF, open reading frame; NSP7, non-structural protein 7; NT-proBNP, N-terminal pro brain natriuretic peptide; RBC, red blood cell; SD, standard deviation; TBIL, total bilirubin; TNF, tumor necrosis factor; PLT, platelet count; eGFR, epidermal growth factor receptor; CRP, C-reactive protein. ↓, significant decrease; ↑, significant increase.

**Table 5:** Enrichment statistical analysis for COVID-19 patients with high antibody responses for S1 IgG, S1-113 IgM, ORF-3a IgG, NSP7 IgG, and S2-97 IgM.

	Severity			Outcome		
Antibody Responses		Fold Enrichments	*p* Value		Fold Enrichments	*p* Value
S1 IgG	Non-critical	0.971	0.635	Survivor	1.021	0.226
	Critical	1.025	0.365	Non-survivor	0.963	0.774
S1-113 IgM	Non-critical	1.639	0.002	Survivor	1.329	0.026
	Critical	0.794	0.998	Non-survivor	0.668	0.874
S2-97 IgM	Non-critical	0.921	0.830	Survivor	1.013	0.355
	Critical	1.069	0.170	Non-survivor	0.858	0.645
ORF 3a IgG	Non-critical	0.870	0.941	Survivor	0.996	0.643
	Critical	1.112	0.059	Non-survivor	1.049	0.357
NSP7 IgM	Non-critical	1.372	0.019	Survivor	0.987	0.766
	Critical	0.852	0.981	Non-survivor	1.144	0.234

**Table 6:** Enrichment statistical analysis for COVID-19 patients with influenza A virus infection.

		Case (n)	Influenza A Virus IgM Antibody			
			Positive	Negative	Fold Enrichments	*p* Value
<41	Mild	20	16	4	1.40	0.00
	Severe	7	3	4	0.79	0.59
41–50	Mild	21	14	7	1.16	0.11
	Severe	16	9	7	1.04	0.33
51–60	Mild	26	15	11	1.01	0.39
	Severe	20	12	8	1.11	0.21
61–70	Mild	27	13	14	0.84	0.81
	Severe	36	22	14	1.13	0.11
71–80	Mild	12	5	7	0.73	0.80
	Severe	21	9	12	0.79	0.82
>80	Mild	4	0	4	0.00	0.79
	Severe	7	3	4	0.79	0.59

## Data Availability

The microarray data of SARS-CoV-2-specific antibody responses were submitted to PMD with the accession PMDE244 [43]. The clinical features of 783 patients are deposited on the COVID-ONE-hi database (www.covid-one.cn) [42].

## Supplementary Materials

The following supporting information can be downloaded at: https://www.mdpi.com/article/10.3390/jcm11195974/s1, Figure S1. (A) The time of onset to sampling for COVID patients of 6 age groups. (B) UMAP analysis of IgG and IgM responses in different age groups. (C) The landscape of SARS-CoV-2 protein antibody responses in different age groups IgG and IgM; Table S1. The chi-square test parameter of severity in association with gender among COVID-19 patients; Table S2. The binary logistic regression parameter of severity and outcome in association with the medical history among COVID-19 patients; Table S3. Enrichment statistical analysis for the patients with high S1 IgG responses.

## References

[1] Huang C., Wang Y., Li X., Ren L., Zhao J., Hu Y., Zhang L., Fan G., Xu J., Gu X. (2020). Clinical features of patients infected with 2019 novel coronavirus in Wuhan, China. Lancet.

[2] Rokni M., Ghasemi V., Tavakoli Z. (2020). Immune responses and pathogenesis of SARS-CoV-2 during an outbreak in Iran: Comparison with SARS and MERS. Rev. Med. Virol..

[3] Ahern D.J., Ai Z., Ainsworth M., Allan C., Allcock A., Angus B., Ansari M.A., Arancibia-Cárcamo C.V., Aschenbrenner D., Attar M. (2022). A blood atlas of COVID-19 defines hallmarks of disease severity and specificity. Cell.

[4] Zhang X., Tan Y., Ling Y., Lu G., Liu F., Yi Z., Jia X., Wu M., Shi B., Xu S. (2020). Viral and host factors related to the clinical outcome of COVID-19. Nature.

[5] Liu Y., Mao B., Liang S., Yang J.W., Lu H.W., Chai Y.H., Wang L., Zhang L., Li Q.H., Zhao L. (2020). Association between age and clinical characteristics and outcomes of COVID-19. Eur. Respir. J..

[6] Mahase E. (2020). COVID-19: Death rate is 0.66% and increases with age, study estimates. BMJ.

[7] Bonanad C., Garcia-Blas S., Tarazona-Santabalbina F., Sanchis J., Bertomeu-Gonzalez V., Facila L., Ariza A., Nunez J., Cordero A. (2020). The Effect of Age on Mortality in Patients with COVID-19: A Meta-Analysis with 611,583 Subjects. J. Am. Med. Dir. Assoc..

[8] Romero Starke K., Reissig D., Petereit-Haack G., Schmauder S., Nienhaus A., Seidler A. (2021). The isolated effect of age on the risk of COVID-19 severe outcomes: A systematic review with meta-analysis. BMJ Glob. Health.

[9] Biswas M., Rahaman S., Biswas T.K., Haque Z., Ibrahim B. (2020). Association of Sex, Age, and Comorbidities with Mortality in COVID-19 Patients: A Systematic Review and Meta-Analysis. Intervirology.

[10] Zimmermann P., Curtis N. (2022). Why Does the Severity of COVID-19 Differ with Age?: Understanding the Mechanisms Underlying the Age Gradient in Outcome Following SARS-CoV-2 Infection. Pediatr. Infect. Dis. J..

[11] Zheng J., Deng Y., Zhao Z., Mao B., Lu M., Lin Y., Huang A. (2022). Characterization of SARS-CoV-2-specific humoral immunity and its potential applications and therapeutic prospects. Cell Mol. Immunol..

[12] Long Q.X., Liu B.Z., Deng H.J., Wu G.C., Deng K., Chen Y.K., Liao P., Qiu J.F., Lin Y., Cai X.F. (2020). Antibody responses to SARS-CoV-2 in patients with COVID-19. Nat. Med..

[13] Ma M.L., Shi D.W., Li Y., Hong W., Lai D.Y., Xue J.B., Jiang H.W., Zhang H.N., Qi H., Meng Q.F. (2021). Systematic profiling of SARS-CoV-2-specific IgG responses elicited by an inactivated virus vaccine identifies peptides and proteins for predicting vaccination efficacy. Cell Discov..

[14] Li Y., Xu Z., Lei Q., Lai D.Y., Hou H., Jiang H.W., Zheng Y.X., Wang X.N., Wu J., Ma M.L. (2021). Antibody landscape against SARS-CoV-2 reveals significant differences between non-structural/accessory and structural proteins. Cell Rep..

[15] Lei Q., Yu C.Z., Li Y., Hou H.Y., Xu Z.W., Yao Z.J., Zhang Y.D., Lai D.Y., Ndzouboukou J.B., Zhang B. (2022). Anti-SARS-CoV-2 IgG responses are powerful predicting signatures for the outcome of COVID-19 patients. J. Adv. Res..

[16] Shankar-Hari M., Vale C.L., Godolphin P.J., Fisher D., Higgins J.P.T., Spiga F., Savovic J., Tierney J., Baron G., The WHO Rapid Evidence Appraisal for COVID-19 Therapies (REACT) Working Group (2021). Association Between Administration of IL-6 Antagonists and Mortality Among Patients Hospitalized for COVID-19: A Meta-analysis. JAMA.

[17] Li X., Xu S., Yu M., Wang K., Tao Y., Zhou Y., Shi J., Zhou M., Wu B., Yang Z. (2020). Risk factors for severity and mortality in adult COVID-19 inpatients in Wuhan. J. Allergy Clin. Immunol..

[18] Santa Cruz A., Mendes-Frias A., Oliveira A.I., Dias L., Matos A.R., Carvalho A., Capela C., Pedrosa J., Castro A.G., Silvestre R. (2021). Interleukin-6 Is a Biomarker for the Development of Fatal Severe Acute Respiratory Syndrome Coronavirus 2 Pneumonia. Front. Immunol..

[19] Investigators R.-C., Gordon A.C., Mouncey P.R., Al-Beidh F., Rowan K.M., Nichol A.D., Arabi Y.M., Annane D., Beane A., van Bentum-Puijk W. (2021). Interleukin-6 Receptor Antagonists in Critically Ill Patients with COVID-19. N. Engl. J. Med..

[20] Jiang H.W., Li Y., Zhang H.N., Wang W., Yang X., Qi H., Li H., Men D., Zhou J., Tao S.C. (2020). SARS-CoV-2 proteome microarray for global profiling of COVID-19 specific IgG and IgM responses. Nat. Commun..

[21] Li Y., Lai D.Y., Lei Q., Xu Z.W., Wang F., Hou H., Chen L., Wu J., Ren Y., Ma M.L. (2021). Systematic evaluation of IgG responses to SARS-CoV-2 spike protein-derived peptides for monitoring COVID-19 patients. Cell Mol. Immunol..

[22] Li Y., Li C.Q., Guo S.J., Guo W., Jiang H.W., Li H.C., Tao S.C. (2020). Longitudinal serum autoantibody repertoire profiling identifies surgery-associated biomarkers in lung adenocarcinoma. EBioMedicine.

[23] Williamson E.J., Walker A.J., Bhaskaran K., Bacon S., Bates C., Morton C.E., Curtis H.J., Mehrkar A., Evans D., Inglesby P. (2020). Factors associated with COVID-19-related death using OpenSAFELY. Nature.

[24] Scully E.P., Haverfield J., Ursin R.L., Tannenbaum C., Klein S.L. (2020). Considering how biological sex impacts immune responses and COVID-19 outcomes. Nat. Rev. Immunol..

[25] Zhang J.J., Cao Y.Y., Tan G., Dong X., Wang B.C., Lin J., Yan Y.Q., Liu G.H., Akdis M., Akdis C.A. (2021). Clinical, radiological, and laboratory characteristics and risk factors for severity and mortality of 289 hospitalized COVID-19 patients. Allergy.

[26] Li Y., Lai D.Y., Zhang H.N., Jiang H.W., Tian X., Ma M.L., Qi H., Meng Q.F., Guo S.J., Wu Y. (2020). Linear epitopes of SARS-CoV-2 spike protein elicit neutralizing antibodies in COVID-19 patients. Cell Mol. Immunol..

[27] Li Y., Ma M.-l., Lei Q., Wang F., Hong W., Lai D.-y., Hou H., Xu Z.-w., Zhang B., Chen H. (2021). Linear epitope landscape of the SARS-CoV-2 Spike protein constructed from 1051 COVID-19 patients. Cell Rep..

[28] Siu K.L., Yuen K.S., Castano-Rodriguez C., Ye Z.W., Yeung M.L., Fung S.Y., Yuan S., Chan C.P., Yuen K.Y., Enjuanes L. (2019). Severe acute respiratory syndrome coronavirus ORF3a protein activates the NLRP3 inflammasome by promoting TRAF3-dependent ubiquitination of ASC. FASEB J..

[29] Gao Y., Yan L., Huang Y., Liu F., Zhao Y., Cao L., Wang T., Sun Q., Ming Z., Zhang L. (2020). Structure of the RNA-dependent RNA polymerase from COVID-19 virus. Science.

[30] Hou H., Zhang B., Huang H., Luo Y., Wu S., Tang G., Liu W., Mao L., Mao L., Wang F. (2020). Using IL-2R/lymphocytes for predicting the clinical progression of patients with COVID-19. Clin. Exp. Immunol..

[31] Han H., Ma Q., Li C., Liu R., Zhao L., Wang W., Zhang P., Liu X., Gao G., Liu F. (2020). Profiling serum cytokines in COVID-19 patients reveals IL-6 and IL-10 are disease severity predictors. Emerg. Microbes Infect..

[32] Chen C., Chen C., Yan J.T., Zhou N., Zhao J.P., Wang D.W. (2020). Analysis of myocardial injury in patients with COVID-19 and association between concomitant cardiovascular diseases and severity of COVID-19. Zhonghua Xin Xue Guan Bing Za Zhi.

[33] Danwang C., Endomba F.T., Nkeck J.R., Wouna D.L.A., Robert A., Noubiap J.J. (2020). A meta-analysis of potential biomarkers associated with severity of coronavirus disease 2019 (COVID-19). Biomark. Res..

[34] Coskun M.E., Alidris A., Temel M.T., Akbayram S., Hizli S. (2019). Plateletcrit: A possible biomarker of inflammation in hepatitis A infection. Niger. J. Clin. Pract..

[35] Nugent J., Aklilu A., Yamamoto Y., Simonov M., Li F., Biswas A., Ghazi L., Greenberg J., Mansour S., Moledina D. (2021). Assessment of Acute Kidney Injury and Longitudinal Kidney Function After Hospital Discharge Among Patients With and Without COVID-19. JAMA Netw. Open.

[36] Liu X., Wang H., Shi S., Xiao J. (2021). Association between IL-6 and severe disease and mortality in COVID-19 disease: A systematic review and meta-analysis. Postgrad. Med. J..

[37] Gao Y., Li T., Han M., Li X., Wu D., Xu Y., Zhu Y., Liu Y., Wang X., Wang L. (2020). Diagnostic utility of clinical laboratory data determinations for patients with the severe COVID-19. J. Med. Virol..

[38] Legros V., Denolly S., Vogrig M., Boson B., Siret E., Rigaill J., Pillet S., Grattard F., Gonzalo S., Verhoeven P. (2021). A longitudinal study of SARS-CoV-2-infected patients reveals a high correlation between neutralizing antibodies and COVID-19 severity. Cell. Mol. Immunol..

[39] Roltgen K., Powell A.E., Wirz O.F., Stevens B.A., Hogan C.A., Najeeb J., Hunter M., Wang H., Sahoo M.K., Huang C. (2020). Defining the features and duration of antibody responses to SARS-CoV-2 infection associated with disease severity and outcome. Sci. Immunol..

[40] Takahashi T., Ellingson M.K., Wong P., Israelow B., Lucas C., Klein J., Silva J., Mao T., Oh J.E., Tokuyama M. (2020). Sex differences in immune responses that underlie COVID-19 disease outcomes. Nature.

[41] Spini A., Giudice V., Brancaleone V., Morgese M.G., De Francia S., Filippelli A., Ruggieri A., Ziche M., Ortona E., Cignarella A. (2021). Sex-tailored pharmacology and COVID-19: Next steps towards appropriateness and health equity. Pharmacol. Res..

[42] Xu Z., Li Y., Lei Q., Huang L., Lai D.Y., Guo S.J., Jiang H.W., Hou H., Zheng Y.X., Wang X.N. (2021). COVID-ONE-hi: The One-stop Database for COVID-19 Specific Humoral Immunity and Clinical Parameters. Genom. Proteom. Bioinform..

[43] Xu Z., Huang L., Zhang H., Li Y., Guo S., Wang N., Wang S.H., Chen Z., Wang J., Tao S.C. (2016). PMD: A Resource for Archiving and Analyzing Protein Microarray data. Sci. Rep..

[44] Ahmed S.I., Hasan S.M.T., Ahmed T. (2020). Obesity is a potential risk factor for COVID-19 associated morbidity and mortality in urban Bangladesh. BMJ.

[45] Bramante C.T., Huling J.D., Tignanelli C.J., Buse J.B., Liebovitz D.M., Nicklas J.M., Cohen K., Puskarich M.A., Belani H.K., Proper J.L. (2022). Randomized Trial of Metformin, Ivermectin, and Fluvoxamine for COVID-19. N. Engl. J. Med..

[46] McCarthy C., O’Donnell C.P., Kelly N.E.W., O’Shea D., Hogan A.E. (2021). COVID-19 severity and obesity: Are MAIT cells a factor?. Lancet Respir. Med..

[47] Popkin B.M., Du S., Green W.D., Beck M.A., Algaith T., Herbst C.H., Alsukait R.F., Alluhidan M., Alazemi N., Shekar M. (2020). Individuals with obesity and COVID-19: A global perspective on the epidemiology and biological relationships. Obes. Rev..

[48] Rodgers G.P., Gibbons G.H. (2020). Obesity and Hypertension in the Time of COVID-19. JAMA.

