# Supplementary material for: Age- and Severity-Associated Humoral Immunity Response in COVID-19 Patients: A Cohort Study from Wuhan, China
Source: J Clin Med. 2022 Oct 10;11(19):5974. doi: 10.3390/jcm11195974 (PMC9571343; doi:10.3390/jcm11195974)
Supplement: Supplementary file 1 [file jcm-11-05974-s001.zip › jcm-1885654-supplementary.pdf]

Supplementary Material

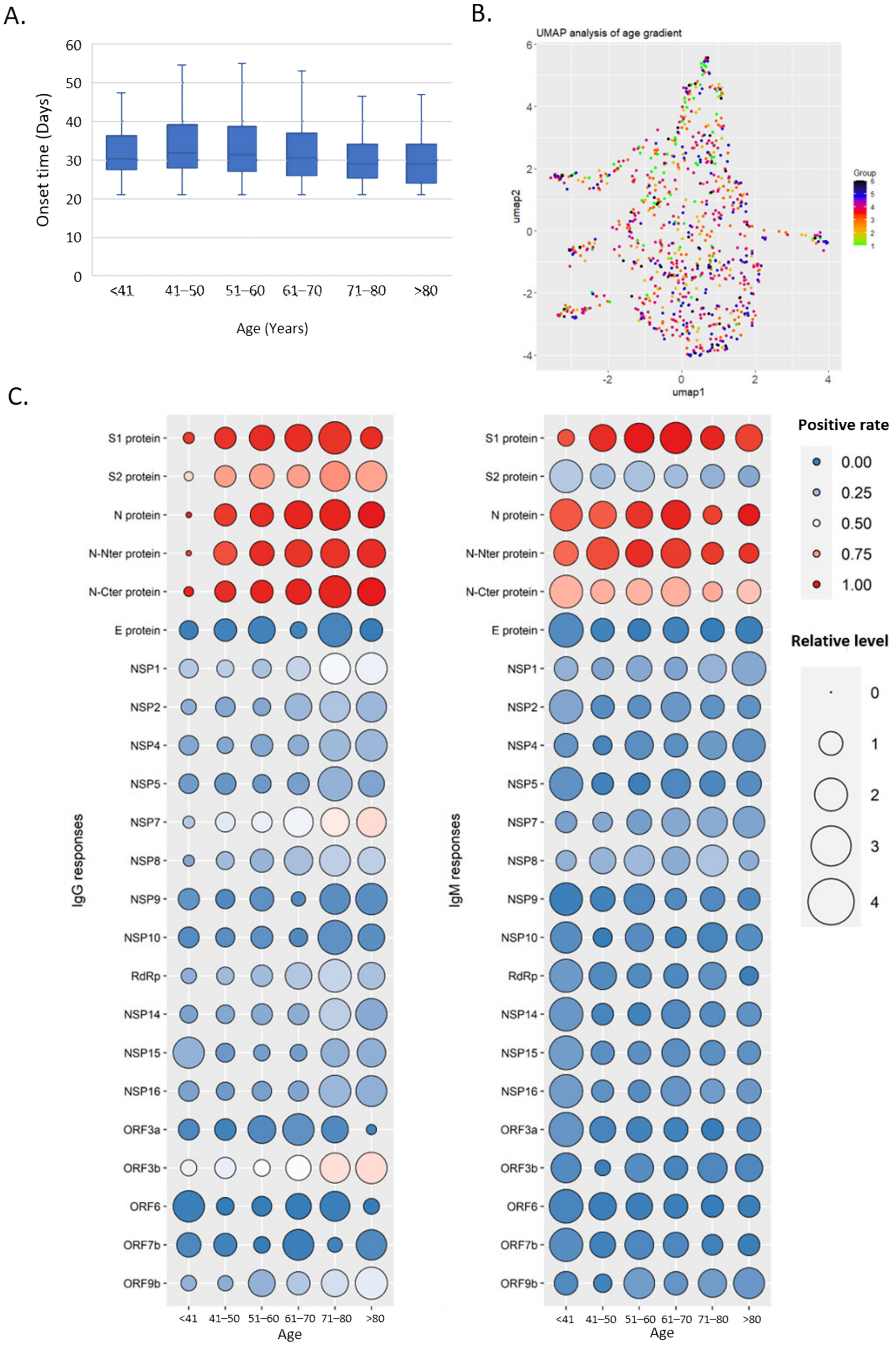

Figure S1. (A) The time of onset to sampling for COVID patients of 6 age groups. (B) UMAP analysis of IgG and IgM responses in different age groups. (C) The landscape of SARS-CoV-2 protein antibody responses in different age groups IgG and IgM.

Table S1. The chi-square test parameter of severity in association with gender among COVID-19 patients.

		Cases (n)	Mild patients, n (%)	Severe patients, n (%)	χ^2	*P*
⩽40	F	52	10 (19.2)	42 (80.8)	0.485	0.486
	M	65	16 (24.6)	49 (75.4)		
41–50	F	56	18 (32.1)	38 (67.9)	1.588	0.208
	M	72	31 (43.1)	41 (56.9)		
51–60	F	116	45 (38.8)	71 (61.2)	7.478	0.006
	M	103	59 (57.3)	44 (42.7)		
61–70	F	166	78 (47.0)	88 (53.0)	10.425	0.001
	M	154	100 (64.9)	54 (35.1)		
71–80	F	104	59 (56.7)	45 (43.3)	7.740	0.005
	M	94	71 (75.5)	23 (24.5)		
> 80	F	34	22 (64.7)	12 (35.3)	0.359	0.549
	M	35	25 (71.4)	10 (28.6)		

Table S2. The binary logistic regression parameter of severity and outcome in association with the medical history among COVID-19 patients.

	Severity					Overcome				
Medical history	*β*	S.E.	Wald χ2	OR (95% CI)	*P*	*β*	S.E.	Wald χ2	OR (95% CI)	*P*
None				1.000					1.000	
Hypertension	0.828	0.139	35.341	2.288 (1.742, 3.006)	0.000	0.036	0.265	0.018	1.036 (0.026, 0.341)	0.893
Diabetes mellitus	0.844	0.176	22.896	2.325 (1.646, 3.285)	0.000	0.281	0.310	0.821	1.325 (0.142, 0.673)	0.365
Respiratory diseases	0.144	0.459	0.098	1.154 (0.470, 2.837)	0.754	0.392	0.766	0.262	1.480 (0.147, 0.628)	0.609
Cardiovascular disease	1.120	0.243	21.330	3.065 (1.905, 4.930)	0.000	0.954	0.334	8.175	2.595 (1.350, 4.991)	0.004
Cancer	1.171	0.462	6.412	3.225 (1.303, 7.982)	0.011	1.309	0.536	5.963	3.701 (1.295, 10.579)	0.015

Table S3. Enrichment statistical analysis for the patients with high S1 IgG responses.

Age	Case (n)	Fold enrichments	p value
<41	5	0.220	1.000
41–50	13	0.503	0.994
51–60	39	1.086	0.549
61–70	71	1.146	0.038
71–80	48	1.438	0.001
>80	10	0.772	0.411
